# Comparative Study Between Citric Acid and Glutaraldehyde in the Crosslinking of Gelatine Hydrogels Reinforced with Cellulose Nanocrystals (CNC)

**DOI:** 10.3390/gels11100790

**Published:** 2025-10-01

**Authors:** Diana Carmona-Cantillo, Rafael González-Cuello, Rodrigo Ortega-Toro

**Affiliations:** Food Packaging and Shelf-Life Research Group (FP&SL), Food Engineering Department, Universidad de Cartagena, Avenida del Consulado Calle 30 No. 48–152, Cartagena de Indias 130015, Colombia; dcarmonac1@unicartagena.edu.co (D.C.-C.); rgonzalezc1@unicartagena.edu.co (R.G.-C.)

**Keywords:** hydrogels, crosslinking agents, cellulose nanocrystals (CNC)

## Abstract

Hydrogels comprise three-dimensional networks of hydrophilic polymers and have attracted considerable interest in various sectors, including the biomedical, pharmaceutical, agricultural, and food industries. These materials offer significant benefits for food packaging applications, such as high mechanical strength and excellent water absorption capacity, thereby contributing to the extension of product shelf life. Therefore, the aim of this study is to compare the performance of citric acid and glutaraldehyde as crosslinking agents in gelatine-based hydrogels reinforced with cellulose nanocrystals (CNC), contributing to the development of safe and environmentally responsible materials. The hydrogels were prepared using the casting method and characterised in terms of their physical, mechanical, and structural properties. The results indicated that hydrogels crosslinked with glutaraldehyde exhibited higher opacity, lower transparency, and greater mechanical strength, whereas those crosslinked with citric acid demonstrated improved clarity, reduced water permeability, and enhanced swelling capacity. The incorporation of CNC further improved mechanical strength, reduced weight loss, and altered both surface homogeneity and optical properties. Microstructural results obtained by SEM were consistent with the mechanical properties evaluated (TS, %E, and EM). The Gel-ca hydrogel displayed the highest elongation value (98%), reflecting better cohesion within the polymeric matrix. In contrast, films incorporating CNC exhibited greater roughness and cracking, which correlated with increased rigidity and mechanical strength, as evidenced by the high Young’s modulus (420 MPa in Gel-ga-CNC2). These findings suggest that the heterogeneity and porosity induced by CNC limit the mobility of polymer chains, resulting in less flexible and more rigid structures. Additionally, the DSC analysis revealed that gelatine hydrogels did not exhibit a well-defined Tg, due to the predominance of crystalline domains. Systems crosslinked with citric acid showed greater thermal stability (higher Tm and ΔH_m_ values), while those crosslinked with glutaraldehyde, although mechanically stronger, exhibited lower thermal stability. These results confirm the decisive effect of the crosslinking agent and CNC incorporation on the structural and thermal behaviour of hydrogels. In this context, the application of hydrogels in packaged products represents an eco-friendly alternative that enhances product presentation. This research supports the reduction in plastic consumption whilst promoting the principles of a circular economy and facilitating the development of materials with lower environmental impact.

## 1. Introduction

Polymers are integral to the production of conventional plastics, which find application in numerous sectors. However, the environmental persistence of these materials, particularly petroleum-derived plastics such as polyethylene, polypropylene, polystyrene, polyethylene terephthalate, and polyvinyl chloride, presents significant concerns. Their resistance to degradation leads to accumulation in terrestrial and marine environments. Furthermore, their fragmentation into microplastics results in the contamination of the food web, thereby posing potential threats to human health [1]. Consequently, there is an increasing interest in the development and utilisation of renewable materials for food packaging applications.

In this regard, hydrogels, which are three-dimensional networks of hydrophilic polymers, have attracted significant attention across various industries, including biomedical, pharmaceutical, agricultural, and food sectors [2]. They offer major advantages for food packaging, such as mechanical strength and a high-water absorption capacity, thereby contributing to the extended shelf-life of food matrices [3]. Furthermore, hydrogels exhibit the ability to encapsulate bioactive compounds, enabling controlled release mechanisms while protecting food products from oxidation and microbial degradation [4]. These materials can be fabricated from different components, such as gelatine and cellulose, further broadening their applications in food packaging.

One of the most extensively studied polymers for the development of these hydrogels is gelatine, which is obtained from collagen when the bonds between the chains are broken or partially hydrolysed. Although gelatine is primarily known for its use as a food ingredient, it also exhibits excellent film-forming properties, which enhance the mechanical strength and barrier characteristics of materials. Moreover, it is widely available and low-cost [5].

Another polymer employed in the manufacture of these materials is cellulose derivatives, such as cellulose nanocrystals (CNC). These nanocrystals are distinguished by their high mechanical strength and excellent barrier properties [6], making them a promising option for food packaging. Their applications include the development of nanocomposites, coatings, and films, all of which contribute to sustainable packaging solutions [7].

On the other hand, the selection of the crosslinking agent influences the characteristics of the material. The most widely used crosslinker at present is glutaraldehyde, a chemical agent that establishes stable covalent bonds with functional groups such as amino and hydroxyl groups, thereby providing hydrogels with greater mechanical strength and stability [8]. However, its potential toxicity in cases where residues are not completely removed represents a limitation. By contrast, citric acid, a natural and non-toxic crosslinker, forms ester bonds with hydroxyl groups, making it a more environmentally friendly and biocompatible option. Although hydrogels obtained with the latter may exhibit lower mechanical strength, their advantage lies in safety and sustainability [9]. Therefore, the aim of this study is to compare the performance of citric acid and glutaraldehyde as crosslinking agents in gelatine-based hydrogels reinforced with cellulose nanocrystals (CNC), thereby contributing to the development of safe and environmentally responsible materials.

## 2. Results and Discussion

### 2.1. Optical and Colour Characteristics

Table 1 details the optical and chromatic characteristics of gelatin-based hydrogels. The materials were chemically modified via crosslinking with either glutaraldehyde or citric acid and subsequently reinforced with cellulose nanocrystals (CNC). The assessment comprised measurements of gloss at 60°, CIE L*a*b* coordinates, hue angle, chroma, and total colour difference, thereby furnishing a comprehensive analysis of the resultant visual appearance.

The characteristics of the formulations analyzed were significantly affected by the type of crosslinking agent applied and the incorporation of cellulose nanocrystals (CNC). In this regard, the Gel-ga and Gel-ga-CNC2 hydrogels exhibited the highest gloss values (94.8 and 97.8, respectively), suggesting highly reflective surfaces. In contrast, Gel-ca-CNC1 and Gel-ca-CNC2 showed the lowest values (31.2 and 33.4), indicating a more matte finish. This effect may be related to the crosslinking capability of glutaraldehyde, which significantly affects the optical behaviour of the hydrogels. This compound forms covalent bonds with the amino groups of gelatins, generating a denser polymeric matrix that alters light dispersion and absorption within the material [10]. Furthermore, the Gel-ga-CNC2 hydrogel contains the highest concentration of cellulose nanocrystals (CNC). These nanocrystals modify light scattering within the material, leading to significant changes in optical properties such as gloss, lightness, and hue [1]. Regarding colour, glutaraldehyde-crosslinked hydrogels (Gel-ga) displayed a reduction in L* values, which quantify the lightness of the hydrogel; lower L* values indicate a visually darker material. This behaviour is attributed to increased polymer network density and the characteristic yellowish tint of glutaraldehyde, which promotes greater light absorption [5]. Additionally, these hydrogels exhibited higher chromatic intensity, evidenced by elevated a* (red) and b* (yellow) values, particularly in Gel-ga-CNC2, which showed the highest chroma (49.4) and a perceptible colour difference (ΔE = 5.2) compared to the control. In contrast, citric acid-crosslinked hydrogels (Gel-ca), especially Gel-ca-CNC1, maintained a lighter and more neutral hue, with low saturation (C = 4.0) and a less intense hue angle (h°). Moreover, the citric acid-crosslinked hydrogels (Gel-ca, Gel-ca-CNC1, Gel-ca-CNC2) exhibited the highest L* values, indicating increased lightness and, therefore, a brighter appearance. They also showed reduced a* and b* values, corresponding to a more neutral tone. These variations may be attributed to the crosslinking mechanism of citric acid, which forms ester bonds with the hydroxyl groups of gelatine, producing a more organised polymeric network with lower optical distortion compared to glutaraldehyde [11]. Although these hydrogels present a more translucent and less intense colour appearance, they also display a cohesive and robust structure, demonstrating that adequate mechanical properties can be achieved without compromising optical quality. This relationship between transparency and robustness makes them particularly attractive for applications requiring a balance between aesthetics, biocompatibility, and functional performance. Regarding colour saturation, the chroma (C) parameter indicated that the Gel-ga-CNC2 formulation had the highest intensity (49.4), reflecting a more saturated colour. Conversely, citric acid-crosslinked hydrogels with CNC, especially Gel-ca-CNC1, exhibited low C values (ranging from 4.0 to 7.3), corresponding to less intense hues. Concerning hue (h), which defines the tint, most samples fell within the yellow–orange range (72–83°). However, Gel-ca-CNC1 showed an atypical value (55.8°), shifting towards more neutral tones. Finally, in terms of total colour difference (ΔE), Gel-ga-CNC1 exhibited the most pronounced variation compared to its control (ΔE = 8.14), whereas Gel-ca-CNC1 and Gel-ca-CNC2 displayed less noticeable changes (ΔE = 5.3 and 1.8, respectively), indicating that these latter formulations minimally alter the visual appearance relative to the reference material.

### 2.2. Transmittance and Opacity

**Figure 1:**
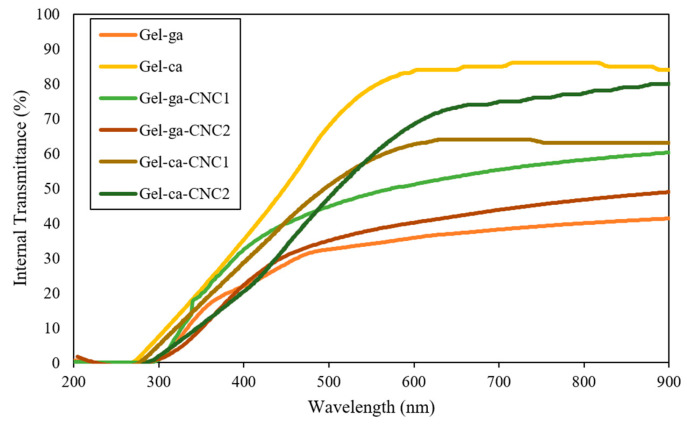
UV–Vis direct transmittance spectra of the different treatments.

Figure 1 presents the UV-Vis transmittance spectra (200–900 nm) for gelatin-based hydrogels crosslinked with glutaraldehyde or citric acid and reinforced with cellulose nanocrystals (CNC). Transmittance measures the amount of light passing through the material, allowing the determination of the transparency of the studied hydrogels [12]. In this context, all materials exhibited low transmission in the ultraviolet region (<300 nm), reflecting high absorption in this range, a typical characteristic of systems with conjugated structures or a high density of organic components. Significant variations in the optical transparency of the hydrogels were observed depending on the crosslinking agent used and the presence of cellulose nanocrystals. Citric acid-crosslinked systems stood out for their exceptional transparency, displaying values above 85% between 600 and 900 nm (Gel-ca sample), indicating minimal interaction with visible radiation. This property was maintained even with the incorporation of CNC (Gel-ca-CNC1 and Gel-ca-CNC2), whose transmittance values ranged from 70 to 80%, confirming that the addition of these nanomaterials does not substantially compromise optical clarity when this crosslinking system is employed [13]. In contrast, glutaraldehyde-crosslinked hydrogels exhibited a marked reduction in transmittance (50–60% in the visible range), particularly in those containing CNC. This decrease is attributed to the formation of crosslinking products that promote light scattering phenomena [12].

**Figure 2:**
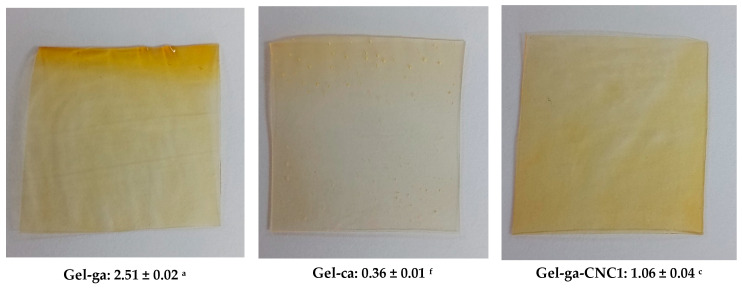

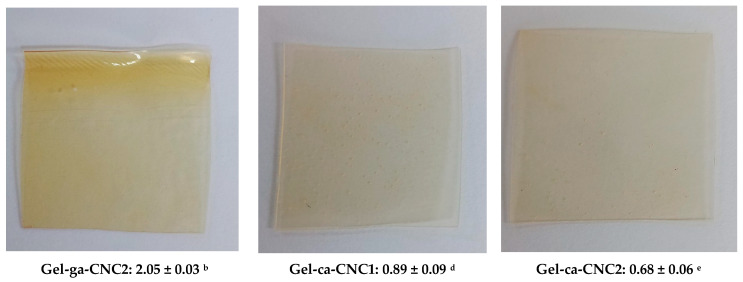
Visual appearance of the films (photographs) and average opacity values ± standard deviation of the analyzed samples. Different superscript letters indicate significant differences (*p* < 0.05) between formulations.

In Figure 2, the opacity values of gelatin-based hydrogels, crosslinked with glutaraldehyde or citric acid and reinforced with cellulose nanocrystals (CNC), are shown. Opacity is defined as the light blocked or absorbed by the film and is inversely related to transmittance [14]. In this context, opacity values show significant differences among the hydrogels depending on the crosslinking agent and the presence of cellulose nanocrystals (CNC). The Gel-ga hydrogel exhibited the highest opacity (2.51 ± 0.02), displaying a yellowish and turbid appearance consistent with its low optical transmittance and high chroma. This is attributed to the formation of dense polymer networks and possible chromophore reactions with glutaraldehyde. This observed behaviour is consistent with the results of [5], wherein UV-Vis spectroscopy revealed pronounced absorption peaks at 200–300 nm and 600 nm upon the introduction of the crosslinking agent. This indicates a marked capacity for light absorption within the hydrogel matrix. The underlying mechanism for this phenomenon is ascribed to a Schiff base reaction, wherein aldehyde groups react with the amino functionalities of CMCS and gelatin, culminating in the generation of a dark yellow pigment. Conversely, Gel-ca displayed the lowest opacity (0.36 ± 0.01) and an almost transparent appearance, resulting from its more open and homogeneous structure. Formulations containing CNC showed intermediate behaviour: Gel-ga-CNC1 (1.06 ± 0.04) and Gel-ca-CNC1 (0.89 ± 0.09), demonstrating that the addition of nanocrystals moderately increases opacity, although this effect is less pronounced when citric acid is used as the crosslinking agent. These results indicate that glutaraldehyde generates higher opacity, whereas citric acid allows the maintenance of high transparency even in the presence of CNC, which is advantageous for applications requiring optical clarity.

### 2.3. Physical Properties and Water Absorption

Table 2 summarises the mean values and corresponding standard deviations for key physical and hygroscopic properties of the hydrogel films. The materials were crosslinked using two distinct agents and reinforced with cellulose nanocrystals (CNC). The parameters assessed encompass film thickness (μm), water vapour transmission rate (WVT), solubility (Hw), moisture content (Xw), and water absorption capacity (WCA).

The thickness of the hydrogels ranged from 196 µm to 259.14 µm. Thickness values showed significant differences (*p* < 0.05) among the hydrogels depending on the crosslinking agent and the presence of CNC. The Gel-ca-CNC2 hydrogel exhibited the greatest thickness (259.14 ± 0.06 µm), whereas Gel-ca-CNC1 was the thinnest (196.00 ± 0.09 µm), which may be related to the CNC concentration directly influencing this parameter. This aligns with the study conducted by [15], which demonstrated that the thickness of edible coatings increased significantly (*p* < 0.05) due to the interaction between CNC concentration and treatment types. On the other hand, Gel-ga-CNC1 and Gel-ga-CNC2 hydrogels, crosslinked with glutaraldehyde, showed significant differences in thickness, with values of 209.14 ± 0.04 µm and 237.28 ± 0.01 µm, respectively. These discrepancies indicate that additional factors including the characteristics or concentration of cellulose nanocrystals (CNC) govern the viscosity of the film-forming solution and modulate its behaviour throughout the drying process [16].

Water vapour permeability (WVT) values also showed significant differences (*p* < 0.05) among the studied formulations. The Gel-ga hydrogel exhibited the highest WVT (1.34 ± 0.08), indicating a less effective barrier against water vapour. Gel-ca-CNC2 recorded the lowest WVT (0.87 ± 0.02), indicating a more compact structure with higher resistance to moisture transfer. However, Gel-ca-CNC1 showed a significantly higher value (1.20 ± 0.03), similar to that of Gel-ga without CNC, demonstrating that in this particular formulation, the CNC did not contribute to improving the barrier property. These results indicate that WVT is not determined solely by the type of crosslinker but also by interactions between components, matrix homogeneity, and the density of the formed polymer network [17].

Significant variations among formulations were evidenced by the statistical differences observed in WCA (*p* < 0.05). Gel-ga-CNC1 showed the highest water absorption capacity (0.55), suggesting higher hydrophilicity, likely due to a less dense structure or greater availability of polar groups within the matrix. Conversely, Gel-ca-CNC2 exhibited the lowest absorption (0.43), consistent with its low water vapour permeability, reinforcing its effectiveness. These results suggest that crosslinking with citric acid was more effective in enhancing barrier properties and reducing water absorption, due to the formation of ester bonds that promote a denser and more hydrophobic polymeric structure. This molecular configuration restricts both water vapour diffusion and moisture retention within the material [18].

Moisture content (Xw) is crucial for maintaining the stability of films used in food packaging. This value differed among the various formulations. Gel-ga exhibited the highest Xw (0.125 ± 0.07), indicating greater water affinity and a less dense network. In contrast, Gel-ca showed a slightly lower value (0.109 ± 0.03), associated with a stronger structure due to citric acid crosslinking. Hydrogels containing CNC displayed relatively high Xw values, primarily due to the hydroxyl groups of the CNC capable of forming hydrogen bonds with water. Additionally, their high surface area promotes moisture adsorption, contributing to increased water content in the matrix [19].

The swelling capacity (Hw) indicates the amount of water the polymeric matrix can absorb without dissolving. Gel-ca recorded the highest Hw (9.43 ± 0.21), reflecting a more porous and flexible structure that facilitates water absorption. In contrast, Gel-ga (3.96 ± 0.18) and Gel-ga-CNC2 (4.00 ± 0.03) exhibited the lowest values, indicating a more compact and rigid matrix with reduced swelling tendency. Samples incorporating CNC, such as Gel-ga-CNC1 and Gel-ca-CNC2, showed intermediate results, suggesting that CNC can reduce expansion by reinforcing the polymer network, although their effect varies depending on the crosslinking agent used [20].

In summary, high WCA, Xw, and Hw values are associated with more open polymer networks, leading to increased WVT. In this regard, Gel-ca-CNC2 exhibited lower values in all these parameters, indicating improved moisture resistance and a more compact structure.

### 2.4. Water and Oil Contact Angles

**Figure 3:**
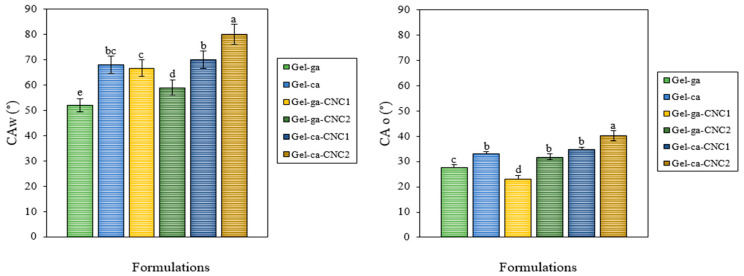
Mean values and associated standard deviations for the water (CAw, °) and oil (CAo, °) contact angles of the investigated films. Different letters indicate significant differences (*p* < 0.05) between formulations.

Figure 3 compares the water contact angles (CAw) and oil contact angles (CAo) of gelatin hydrogels crosslinked with glutaraldehyde or citric acid and reinforced with cellulose nanocrystals (CNC). These values allow the assessment of the hydrophilic or hydrophobic character of the hydrogels, which is influenced by the type of crosslinking agent and the incorporation of CNC. The contact angle results show significant differences (*p* < 0.05) between formulations, depending on the crosslinking agent used and the presence of CNC. Hydrogels crosslinked with citric acid, particularly Gel-ca-CNC2, exhibited the highest CAw values (80°), indicating a less water-adherent surface. Conversely, Gel-ga showed the lowest CAw (52°), suggesting higher hydrophilicity. This phenomenon may be attributable to residual polar functional groups for instance, hydroxyl and carbonyl moieties which retain surface accessibility following the crosslinking process. Moreover, the network formed by glutaraldehyde could create a more porous or less dense structure, facilitating interaction with water [21]. In contrast, citric acid forms more compact ester bonds, limiting the exposure of polar groups and resulting in higher contact angles, indicative of a more hydrophobic character [22]. Additionally, crosslinking with CNC, particularly in the presence of citric acid, appears to slightly increase resistance to oil contact, which may be desirable in applications such as food packaging. Regarding the oil contact angle (CAo), Gel-ga-CNC1 and Gel-ca-CNC2 exhibited the highest oleophobicity. In contrast, Gel-ga and Gel-ca-CNC1 demonstrated greater affinity for oils. These findings indicate that both the incorporation of CNC and the choice of crosslinking agent are key factors in determining surface wetting properties. This tunable capacity is crucial for the development of functional films, such as coatings or packaging materials with water- or oil-repellent properties, depending on the intended application.

### 2.5. Cumulative Weight Loss

**Figure 4:**
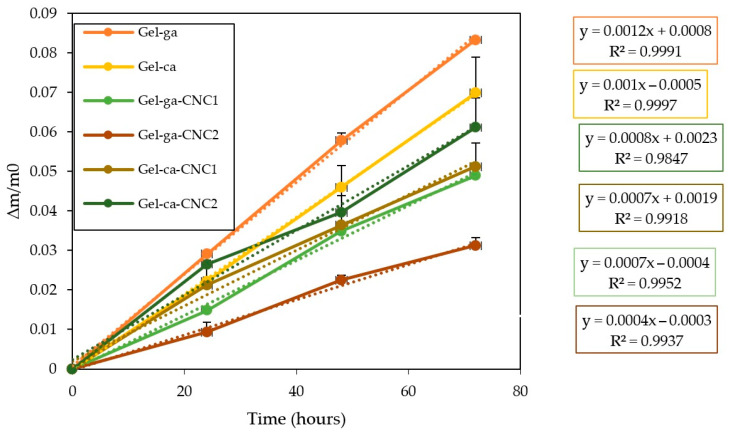
Cumulative mass loss of the investigated films under ambient storage conditions (25 °C). Trendlines are denoted by dashed curves.

Figure 4 displays the cumulative mass loss of gelatin-based hydrogels that were crosslinked using either glutaraldehyde or citric acid and subsequently reinforced with cellulose nanocrystals (CNC). It is evident that the control formulations exhibited the highest rates of weight loss according to their fitted equations. This makes them more prone to material degradation and indicates a structure with greater affinity for water. Consequently, water diffusion through the film is facilitated, leading to the leaching of soluble components [23]. Additionally, hydrogels containing cellulose nanocrystals (CNC) showed reduced weight loss, with Gel-ga-CNC1 and Gel-ga-CNC2 displaying the lowest values. This behaviour suggests that CNC reinforce the polymer matrix, limiting water penetration and thereby reducing material degradation [24].

### 2.6. Mechanical Properties

Table 3 presents the mean values and corresponding standard deviations for the mechanical properties of the investigated hydrogel films: Elastic Modulus (EM), Tensile Strength (TS), and Percentage Elongation at Break (E). These properties indicate the resistance and flexibility of the materials. The TS values represent the maximum strength of the hydrogels before breaking. Hydrogels with the highest concentration of CNC exhibited the highest TS values, particularly Gel-ga-CNC2 (42.2 ± 2.1), indicating greater strength compared to hydrogels without CNC. This suggests that higher CNC content promotes the formation of a more compact network and better load transfer within the matrix, thereby reinforcing the structural integrity of the hydrogels [25]. The crosslinking agent also influenced the mechanical behaviour: hydrogels crosslinked with glutaraldehyde showed greater tensile strength than those crosslinked with citric acid. This is likely due to the formation of more rigid covalent bonds with glutaraldehyde, in contrast to the ester bonds formed by citric acid [26]. The Young’s modulus (EM), a key indicator of material stiffness, ranged from 340 to 420 MPa, demonstrating that both the type of crosslinker and the addition of CNC affected hydrogel rigidity. Unreinforced formulations (Gel-ga and Gel-ca) recorded the lowest moduli (350 and 340 MPa, respectively). In contrast, CNC-reinforced hydrogels (Gel-ga-CNC2 and Gel-ca-CNC2) showed a significant increase (420 and 405 MPa), confirming that cellulose nanocrystals promote a more compact and rigid internal structure. Regarding elongation (E), all hydrogels exhibited high deformability before rupture, with values ranging from 83% to 98%. Unreinforced hydrogels (Gel-ga and Gel-ca) achieved the highest percentages (96% and 98%, respectively), indicating that their polymer networks have greater freedom to stretch, resulting in high flexibility. This behaviour is consistent with [27], where unreinforced hydrogels without CNC exhibited the highest elongation values. On the other hand, CNC-reinforced hydrogels, Gel-ga-CNC2 and Gel-ca-CNC2, showed a slight decrease in elongation (83% and 88%, respectively). This reduction suggests that the incorporation of CNC partially restricts polymer chain mobility, increasing stiffness but slightly reducing deformability. Overall, the incorporation of cellulose nanocrystals (CNC) had a notable impact on the mechanical properties of the hydrogels, reflected in increased stiffness (EM) and tensile strength (TS). Additionally, elongation (E) remained relatively stable across all formulations, indicating that the materials maintain adequate flexibility despite structural reinforcement.

### 2.7. Microstructure

**Figure 5:**
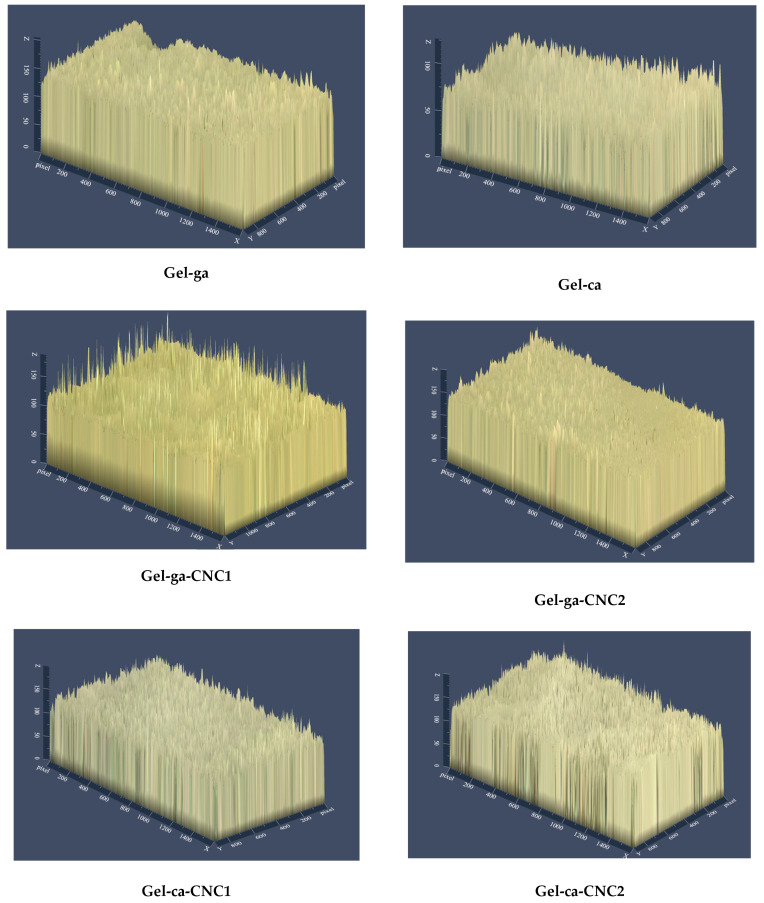
2.5D surface optical micrographs (10×) of the biodegradable hydrogel studied.

In Figure 5, the 2.5D micrographs show differences (*p* < 0.05) in the surface topography among the formulations, depending on the crosslinking agent used and the presence of cellulose nanocrystals (CNC). The hydrogels (Gel-ga-CNC1 and Gel-ca) exhibited rougher and more heterogeneous surfaces. In these two formulations, regions with lighter tones were also observed, indicating significant topographical irregularities and an increase in surface roughness. This phenomenon could be attributed to a heterogeneous molecular structure resulting from irregular crosslinking processes or poor compatibility within the polymeric matrix [28]. Conversely, the hydrogels Gel-ga-CNC1 and Gel-ca-CNC1 showed smoother and more uniform surfaces. This greater uniformity may be related to a more organized structure, which benefits barrier properties. Overall, the incorporation of CNC and the type of crosslinking agent directly influence the surface microstructure and functional performance of the material. This aligns with the findings reported by [29], which state that the high density of hydroxyl groups (-OH) on the surface of cellulose nanocrystals (CNC) promotes their aggregation in aqueous media through interparticle hydrogen bonding, forming clusters that are difficult to disperse. This tendency to agglomerate limits their uniform distribution in polymeric matrices. However, surface modification of CNC allows optimization of interfacial interactions in the materials. These results are consistent with the gloss data measured at 60°, where formulations with smoother surfaces, such as Gel-ga (94.80) and Gel-ga-CNC2 (97.80), exhibited higher reflectance. In contrast, those with greater roughness and heterogeneity, such as Gel-ca-CNC1 (31.29) and Gel-ca-CNC2 (33.43), showed significantly lower values. Thus, the correlation between roughness and gloss demonstrates that both the crosslinking agent and CNC distribution directly affect the surface microstructure and, consequently, the optical properties of the material.

**Figure 6:**
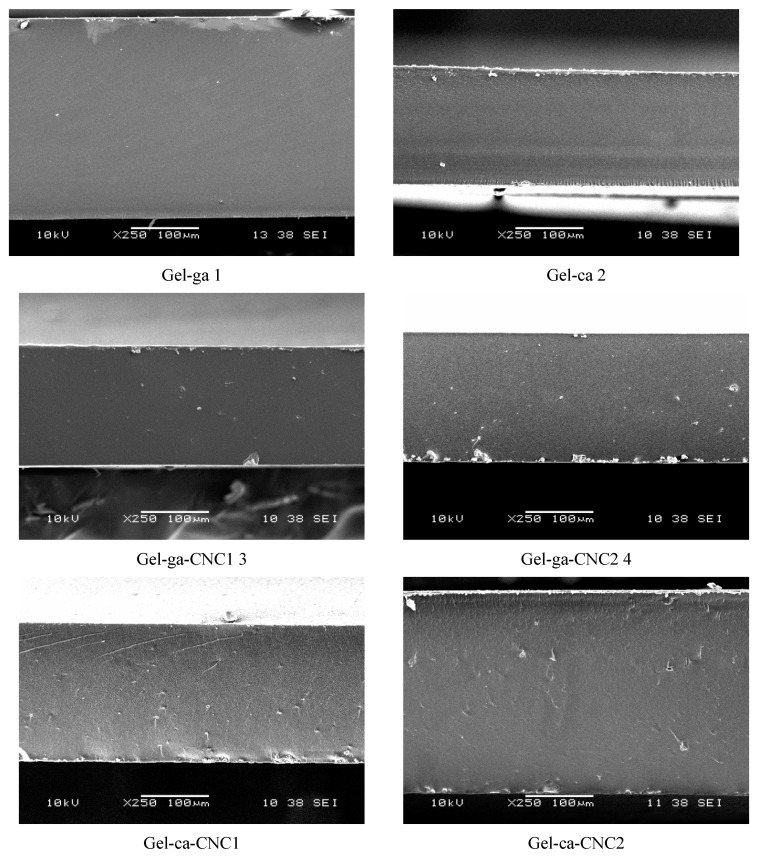
SEM micrographs of the cross-sections of the films studied, observed at 250×.

Figure 6 shows the microstructural analysis of the hydrogels obtained using a scanning electron microscope at 250× magnification. The SEM images reveal significant differences depending on the concentration of cellulose nanocrystals (CNC) and the type of crosslinking agent employed. In this regard, the Gel-ga hydrogel tends to exhibit fewer fractures and pores, which may be attributed to the strong crosslinking capacity of glutaraldehyde, forming a denser and more resistant network due to its high reactivity with the amino groups of gelatins [30]. In contrast, the Gel-ca hydrogel displays a more cracked and porous texture, which could be related to the fact that citric acid, by generating less efficient crosslinks or requiring more controlled conditions, results in a less cohesive structure that is more prone to fracture during drying and observation [30]. On the other hand, the addition of CNC led to a slight modification in the surface texture of the samples, more evident in Gel-ca-CNC1 and Gel-ca-CNC2, where the surface appears rougher and more irregular. This may indicate increased porosity or the formation of microdomains induced by the physical interaction between gelatin and CNC. In the systems crosslinked with glutaraldehyde and CNC (Gel-ga-CNC1 and Gel-ga-CNC2), the network appears more compact and homogeneous, suggesting that the presence of CNC does not negatively interfere with network formation, but may even reinforce it without inducing fractures [31].

**Figure 7:**
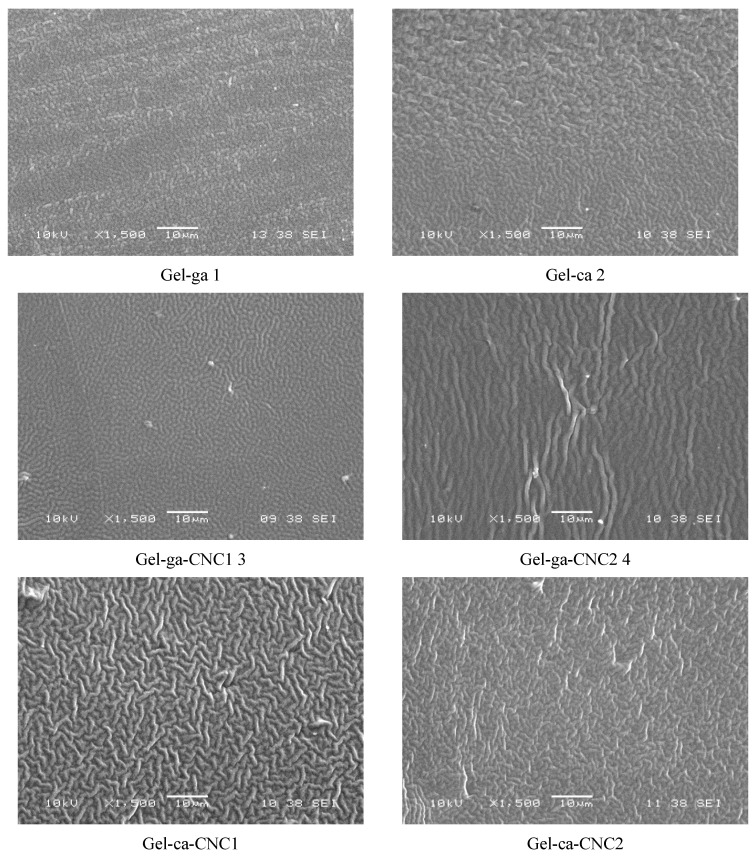
SEM micrographs of the cross-sections of the films studied, observed at 1500×.

Figure 7 shows the SEM micrographs of the cross-sections of the hydrogels obtained, where microstructural differences between the samples can be observed. The control hydrogels (Gel-ga and Gel-ca) exhibited relatively homogeneous microstructures, although Gel-ca displayed more pronounced roughness, which may be associated with a less ordered organisation of the protein network. In contrast, the incorporation of cellulose nanocrystals (CNC) substantially altered the microstructure, promoting more compact configurations. In particular, Gel-ga-CNC1 and Gel-ga-CNC2 exhibited a uniform dispersion of the nanoparticles, suggesting good compatibility between the polymer and the nanoscale reinforcements. The Gel-ca-CNC2 hydrogel, crosslinked with citric acid, showed regions of agglomeration and slight porosity, indicating that at high CNC concentrations the protein–nanocrystal interaction becomes less effective. The additional crosslinking restricts the mobility of the gelatine chains, hindering the homogeneous dispersion of CNC and promoting the formation of agglomerated microdomains within the matrix [32].

Considering the mechanical properties, the SEM microstructural results are consistent with the values obtained for tensile strength (TS), elongation (%E), and Young’s modulus (EM). The highest elongation value (98%) observed for the Gel-ca hydrogel indicates better cohesion within the polymeric matrix. In contrast, the greater roughness and cracking observed in the films containing cellulose nanocrystals are associated with increased rigidity and mechanical strength, as evidenced by the higher Young’s modulus (420 MPa in Gel-ga-CNC2). The reduced flexibility in these formulations suggests that the more heterogeneous and porous structure hinders the mobility of the polymer chains, resulting in stiffer films [32].

In summary, while the mechanical and microstructural analyses demonstrate the effectiveness of glutaraldehyde as a crosslinking agent in reinforcing gelatine hydrogels with cellulose nanocrystals, its potential cytotoxicity cannot be overlooked. Residual glutaraldehyde may pose health risks if not completely removed, as reported in previous in vitro and in vivo studies, which indicate possible cytotoxic and mutagenic effects [33]. In contrast, citric acid, a natural and non-toxic crosslinker, offers a safer and environmentally friendly alternative, albeit with slightly lower mechanical performance. These findings highlight the importance of considering both the functional properties and the biocompatibility of crosslinking agents in the development of hydrogels for biomedical and packaging applications, supporting the pursuit of materials that are both effective and safe.

### 2.8. Thermal Properties (DSC Thermograms)

**Figure 8:**
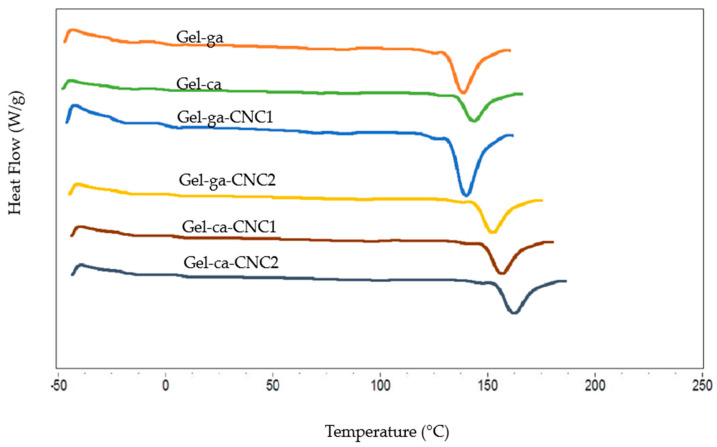
DSC thermograms of gelatin hydrogels with different crosslinkers and CNC contents.

In Figure 8, the differential scanning calorimetry (DSC) curves corresponding to gelatine hydrogels, prepared with different crosslinking agents and cellulose nanocrystal (CNC) concentrations, are presented. Table 4 details the melting temperature and enthalpy values (Tm and ΔH_m_, respectively) obtained from these thermograms.

The DSC thermograms display a single endothermic peak between 149 and 175 °C, associated with the melting of the crystalline domains of the hydrogels. Moreover, no detectable glass transition (Tg) was observed under the experimental conditions employed, which can be attributed to the predominance of crystalline domains [34]. The results reveal a remarkable difference in thermal stability depending on the crosslinking agent. The formulation Gel-ca-CNC2 records the highest melting temperature (174.6 °C), highlighting a synergistic effect in which CNCs, in combination with citric acid, reinforce the polymeric network and promote exceptionally stable crystalline domains [35]. Conversely, in the glutaraldehyde-based matrix, the incorporation of CNC1 reduces the initial Tm (149.7 °C), possibly due to interference in chain organisation. Nevertheless, this stability is recovered at higher CNC concentration (Gel-ga-CNC2, 169.5 °C), demonstrating the crucial influence of dosage. The addition of cellulose nanocrystals (CNC1 and CNC2) shifts the peaks towards higher temperatures, confirming that CNCs act as reinforcing agents that enhance the formation of more stable crystalline regions resistant to melting. This is consistent with the findings of [36], where CNC incorporation increased the crystalline regions of PVA films. The degree of crystallinity of the composite can be significantly enhanced through the introduction of hydrogen bonding systems. Regarding the melting enthalpy (ΔH_m_), the highest values were recorded in the Gel-ca (304.8 J/g) and Gel-ca-CNC2 (277.9 J/g) formulations. This finding confirms that citric acid as a crosslinking agent generates a system with a higher degree of structural order and a more energetically stable network of interactions [9]. In contrast, the addition of CNCs into glutaraldehyde networks resulted in a significant decrease in ΔH_m_ (in the range of 235.9–238.1 J/g), indicating the formation of a less homogeneous crystalline network.

A correlation between the mechanical and thermal properties is evident. Films crosslinked with glutaraldehyde exhibit superior mechanical strength, attributable to the formation of a rigid polymeric network through covalent bonds. However, this structural rigidity restricts chain mobility, resulting in lower melting temperature and enthalpy (Tm and ΔH_m_) in the DSC analyses. On the other hand, hydrogels crosslinked with citric acid, despite exhibiting inferior mechanical performance, demonstrate greater thermal stability (higher Tm and ΔH_m_ values).

## 3. Conclusions

The incorporation of cellulose nanocrystals (CNC) and the choice of crosslinking agents directly influence the mechanical, optical, and physical properties of the resulting hydrogels. Hydrogels crosslinked with glutaraldehyde exhibited higher opacity, lower transparency, and greater mechanical strength, whereas those crosslinked with citric acid showed improved clarity, lower water permeability, and greater swelling capacity. The addition of CNC contributed to enhanced mechanical strength, reduced weight loss, and modifications in both surface homogeneity and optical properties. Consequently, the selection of the crosslinking agent in combination with CNC reinforcement enables the tailoring of hydrogel properties for specific applications, achieving an optimal balance between transparency, flexibility, and moisture resistance. The microstructural results obtained by SEM are consistent with the mechanical properties evaluated (TS, %E, and EM). The Gel-ca hydrogel exhibited the highest elongation value (98%), reflecting better cohesion within the polymeric matrix. In contrast, films incorporating CNC displayed greater roughness and cracking, which correlated with increased rigidity and mechanical strength, as demonstrated by the high Young’s modulus (420 MPa in Gel-ga-CNC2). These findings suggest that the heterogeneity and porosity induced by CNC limit the mobility of the polymer chains, resulting in less flexible and more rigid structures. The calorimetric analysis (DSC) established that hydrogels crosslinked with citric acid exhibited greater thermal stability, as reflected in their higher melting temperature and enthalpy values (Tm and ΔH_m_). In contrast, the glutaraldehyde-based formulations demonstrated enhanced mechanical resistance, although associated with lower thermodynamic stability. These findings highlight the decisive influence of both the type of crosslinking agent and the concentration of cellulose nanocrystals (CNC) in tailoring the structural, thermal, and mechanical properties of gelatine-based hydrogels. The versatility of these hydrogels makes them a sustainable alternative for a wide range of packaging applications while also improving product aesthetics. Accordingly, this work not only contributes to reducing plastic consumption but also supports the transition towards a circular economy through the development of low-environmental-impact materials.

## 4. Materials and Methods

### 4.1. Materials

Food-grade gelatine was sourced from local retail suppliers in Cartagena, Colombia. Cellulose nanocrystals (CNC) were procured from CelluForce (Montreal, QC, Canada). The chemical reagents glycerol, citric acid, and glutaraldehyde—were acquired from PANREAC (Bogotá, Colombia).

### 4.2. Hydrogel Preparation

**Figure 9:**
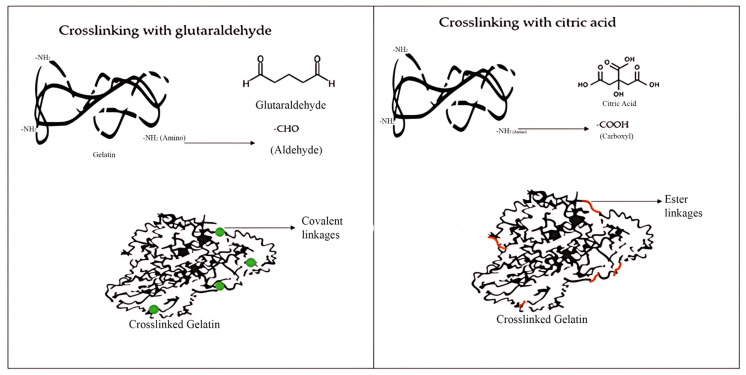
Crosslinking reaction between gelatine with glutaraldehyde and citric acid.

The hydrogel synthesis was adapted from the methodology outlined by [27]. As detailed in Table 5, various formulations were investigated. These were prepared by dissolving gelatine and cellulose nanocrystals (CNC) in water under constant agitation at 50 °C. The CNC concentrations were set at 2% and 4% relative to the gelatine mass, based on values reported in the literature for similar systems. Levels below 2% have been shown to provide only limited reinforcement, whereas concentrations above 6–8% may promote agglomeration and impair the homogeneity and transparency of the films. Accordingly, the selected concentrations represent an intermediate range suitable for assessing the balance between mechanical enhancement and structural stability of the hydrogels [37]. Following homogenisation, glycerol was incorporated as a plasticiser at a concentration of 15% (*w*/*w*, relative to the biopolymer mass), and the mixture was maintained under agitation for 15 min. Subsequently, glutaraldehyde or citric acid was added dropwise as the crosslinking agent, initiating the reticulation reaction: in the case of glutaraldehyde, through the formation of covalent bonds between the amino groups of gelatine and the aldehyde groups; and in the case of citric acid, via esterification reactions between the hydroxyl groups of gelatine/CNC and the carboxyl groups of the acid (See Figure 9). The mixture was then stirred for a further 4 h before being oven-dried at 50 °C.

### 4.3. Characterisation of the Films

#### 4.3.1. Colour

The surface colour of the films was assessed with a portable colourimeter (CHN Spec CS-10) to determine the CIE L*a*b* coordinates, hue angle (h°), and chroma (C). Within the CIE L*a*b* colour space, the L* axis represents lightness, whilst the a* and b* axes denote colour directions (e.g., red-green and yellow-blue, respectively). The colour was then benchmarked against a reference sample to quantify the chromatic variation. This involved calculating the differential values for each coordinate (ΔL*, Δa*, and Δb*). The total colour difference (ΔE) was subsequently computed employing the standard equation.

(1) $$∆E^{*}=\sqrt{{∆L}^{*2}+{∆b}^{*2}+{∆a}^{*2}}$$

#### 4.3.2. Gloss

Gloss measurements were conducted at a 60° angle in accordance with the standard method outlined by [38]. The analysis was performed using a flat-surface glossmeter (3NH YG268 multi-angle glossmeter, Minolta, Langenhagen, Germany). A total of five film samples were assessed, with seven replicate measurements taken per sample. The results are reported in Gloss Units (GU).

#### 4.3.3. Transmittance and Opacity

The analytical procedure was adapted from the method of [39]. The transmittance of the film samples was measured across the UV-visible spectrum employing a BIOBASE BK-UV1900 spectrophotometer (Biobase Biodustry Co., Ltd., Jinan, China). For analysis, the films were sectioned into rectangular strips (1 cm × 3 cm) and affixed to the interior surface of the spectrophotometer’s quartz cuvette. The opacity was subsequently calculated utilising Equation (2).

(2) $$\mathrm{Opacity}=\frac{\mathrm{Absorbance}\;(600\;\mathrm{nm})}{\mathrm{Thickness}\;(\mathrm{mm})}$$

#### 4.3.4. Thickness

Film thickness was gauged with a digital micrometre (TOP EU TL268, HongKong Proster Trading Limited, Hong Kong, China). Seven measurements were taken at random positions across the film surface, and the mean value along with the corresponding standard deviation were subsequently calculated.

#### 4.3.5. Moisture Content (Xw) and Swelling Degree (Hw) of the Hydrogels

The water absorption capacity was determined according to the method adapted from [39]. Film specimens (2 × 2 cm) were initially weighed to establish their dry mass (*W*_1_). They were subsequently oven-dried at 60 °C to constant weight (*W*_2_), then immersed in 25 mL of distilled water at ambient temperature. Following a 24 h immersion period, the water was decanted, and the samples were superficially dried with filter paper prior to a final weighing (*W*_3_).

(3) $$\mathrm{Moisture}\;\mathrm{content}=\frac{W1-W2}{W1}$$

(4) $$\mathrm{Swelling}=\frac{W3-W2}{W3}$$

#### 4.3.6. Water Absorption Capacity (WCA)

The water absorption capacity was ascertained in accordance with the protocol established by [1]. Specimens (2 × 2 cm) were conditioned in a desiccator over calcium chloride to maintain 0% relative humidity at ambient temperature. Mass measurements were recorded at 24 h intervals until equilibrium was achieved, denoting the dry mass (*W_s_*). Subsequently, the specimens were transferred to a second desiccator containing a saturated potassium sulphate solution to maintain a high-humidity environment. Mass measurements were continued at 24 h intervals until a stable weight was attained, designated as the hydrated mass (*W_h_*). The water absorption capacity was subsequently computed employing the following equation.

(5) $$\mathrm{Absorption}\;\mathrm{capacity}\;(\%)=\frac{W_{h}-W_{s}}{W_{s}}\times 100\%$$

#### 4.3.7. Water Vapour Permeability (WVT)

Water vapour permeability (WVT) was ascertained via a gravimetric technique, adapting the method of [40]. A humidity gradient was established from 52.8% to 100% relative humidity (RH) at 25 °C. Intact films, free from physical defects, were selected for testing. Payne permeability cups, filled with distilled water to generate 100% RH on one film surface, were employed. The assemblies were placed in environmental cabinets maintained at 25 °C and 52.8% RH using oversaturated magnesium nitrate solutions. To simulate conditions relevant for packaging high water-activity products, the film’s external surface was exposed to this lower humidity. Mass measurements of the cups were taken at regular intervals using an analytical balance (±0.0001 g). Upon reaching steady-state conditions, the water vapour transmission rate (WVT) was calculated from the slope of the mass loss versus time regression line, normalised by the film’s exposed area. The analysis was conducted in triplicate, with results expressed as the mean ± standard deviation.

#### 4.3.8. Water and Oil Contact Angle

A film specimen (2 × 2 cm) was mounted on a horizontal white background. A single droplet of distilled water (with dye) or palm oil was then deposited onto its surface. Following a 30 s dwell time, an image was acquired using a digital camera, ensuring the lens was maintained at a fixed distance of 20 cm from the sample. The contact angle was subsequently determined through analysis of the captured image utilising Gonio trans software version 1.0.3. The assay was performed in triplicate for each formulation, and the mean contact angle value was recorded [41].

#### 4.3.9. Cumulative Weight Loss

The water sorption kinetics were evaluated according to an adapted methodology based on [42]. Film specimens (2 × 2 cm) were initially conditioned in a desiccator over calcium chloride to maintain 0% relative humidity at ambient temperature. Mass measurements were recorded at 24 h intervals until equilibrium was attained (72 h), denoting the dry mass (*W_s_*). The specimens were then transferred to a second desiccator containing a saturated potassium sulphate solution to maintain a high-humidity environment. Mass measurements continued at 24 h intervals until a stable mass was achieved (72 h), designated as the hydrated mass (*W_h_*). Sorption kinetics were plotted as cumulative mass change versus time for each formulation, where each data point represented the total change from the initial dry mass at the given interval (see Equation (6)). The experiment was conducted in triplicate.

(6) $$\mathrm{Cumulative}\;\mathrm{loss}=P_{0}-P_{t}$$

$$P_{0}=\mathrm{Initial}\;\mathrm{weight}\;\mathrm{of}\;\mathrm{the}\;\mathrm{hydrogel}$$

$$P_{t}=\mathrm{Initial}\;\mathrm{weight}\;\mathrm{of}\;\mathrm{the}\;\mathrm{films}\;\mathrm{at}\;\mathrm{the}\;\mathrm{time}\;\mathrm{interval}$$

#### 4.3.10. Mechanical Properties

The mechanical properties of the films were characterised in accordance with the method described by [43]. The parameters assessed included the elastic modulus (EM), tensile strength (TS), and percentage elongation at break (E). Measurements were performed using a texture analyser (TX-700 TEXTURE ANALYZER, Lamy Rheology SARL, Champagne-au-Mont-d’Or, France) equipped with a 500 N load cell, at a crosshead speed of 50 mm/min. Specimens, measuring 1 cm in width and 100 cm in length, were tested with an initial grip separation of 5 cm.

#### 4.3.11. Microstructure

The surface microstructure of the film specimens was examined using an optical microscope (ZEISS Model 415500-1800-000, Carl Zeiss/ZEISS, Oberkochen, Germany) at a 10× magnification. For this analysis, 1 cm × 1 cm samples were prepared to facilitate observation of the surface topography. Prior to characterisation, all samples were conditioned for one week at 25 °C and 52.8% relative humidity [44]. The cross-sectional microstructure was examined using a scanning electron microscope (JEOL JSM-5910, JEOL Ltd., Akishima, Japan). For this purpose, the samples were dried in desiccators containing P_2_O_5_ for two weeks to ensure complete dehydration. Subsequently, 0.5 cm sections of the films were cut, mounted on copper stubs, sputter-coated with gold, and observed at 10 kV.

#### 4.3.12. Thermal Properties (DSC Thermograms)

The thermal characterisation of the hydrogels was carried out using a Pyris differential scanning calorimeter (DSC Q2000, TA Instruments, New Castle, DE, USA). For the analysis, hydrated samples between 5 and 10 mg were hermetically sealed in aluminium pans, with an empty pan used as a reference. The tests were performed within a temperature range of −50 °C to 250 °C, at a heating rate of 5 °C per minute and under a nitrogen flow of 50 mL/min. The protocol consisted of two consecutive heating and cooling cycles to eliminate any effect of previous thermal history, thereby enabling an accurate determination of the glass transition temperature (Tg) and the endothermic events associated with water loss from the polymer matrix [4,45].

### 4.4. Statistical Analysis

Statistical analysis was performed using one-way analysis of variance (ANOVA). Where significant differences were identified (*p* < 0.05), Fisher’s least significant difference (LSD) post hoc test was applied for multiple comparisons between sample means. All statistical computations were conducted with Statgraphics Centurion 19 software, version 19.7.01 (Manugistics Corp., Rockville, MD, USA).

## Figures and Tables

**Table 1:** Average values and standard deviations of gloss (GU) and colour parameters, including lightness (L), red/green coordinate (a), yellow/blue coordinate (b), chroma (C), and hue angle (h, °), for the analyzed film.

Formulation	Gloss at 60°	Colour Parameters					ΔE	CIE Lab Colour
		L*	a*	b*	C	h		
Gel-ga	94.80 ± 3.89 ^a^	80.7 ± 0.7 ^c^	4.4 ± 0.1 ^e^	32.6 ± 0.2 ^b^	25.9 ± 0.2 ^b^	82.8 ± 0.1 ^a^	-	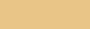
Gel-ca	45.29 ± 1.11 ^c^	87.4 ± 0.6 ^ab^	2.0 ± 0.2 ^d^	7.9 ± 0.4 ^e^	8.8 ± 1.2 ^c^	78.5 ± 2.8 ^c^	-	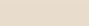
Gel-ga-CNC1	78.60 ± 1.43 ^b^	77.3 ± 0.5 ^b^	7.8 ± 1.21 ^a^	47.4 ± 0.5 ^c^	48.8 ± 0.4 ^c^	80.0 ± 0.7 ^b^	8.14 ± 0.3 ^a^	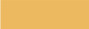
Gel-ga-CNC2	97.80 ± 2.89 ^d^	76.3 ± 2.9 ^b^	10.36 ± 2.5 ^b^	48.31 ± 0.9 ^a^	49.4 ± 2.7 ^a^	77.9 ± 1.17 ^b^	5.2 ± 0.5 ^b^	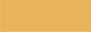
Gel-ca-CNC1	31.29 ± 1.89 ^e^	89.9 ± 0.5 ^a^	2.40 ± 0.1 ^c^	3.30 ± 0.3 ^f^	4.00 ± 0.1 ^c^	55.8 ± 2.9 ^e^	5.3 ± 0.9 ^b^	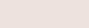
Gel-ca-CNC2	33.43 ± 1.40 ^d^	85.7 ± 0.7 ^a^	2.50 ± 0.1 ^c^	8.4 ± 0.2 ^d^	8.7 ± 0.2 ^ab^	72.6 ± 1.3 ^d^	1.8 ± 0.4 ^c^	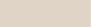

Distinct superscript letters denote statistically significant differences (*p* < 0.05) between the formulations.

**Table 2:** Mean values and standard deviations for the following properties of the investigated films: thickness (μm), water vapour transmission rate (WVT, g·mm/kPa·h·m^2^), water absorption capacity (WCA, g water/g dry film), moisture content (Xw, g water/g dry film), and solubility/swelling (Hw, g water absorbed/g dry film).

Formulations	Thickness	WVT	WCA	Xw	Hw
Gel-ga	228.14 ± 0.035 ^b^	1.34 ± 0.08 ^a^	0.46 ± 0.06 ^c^	0.125 ± 0.07 ^c^	3.96 ± 0.18 ^c^
Gel-ca	207.71 ± 0.077 ^b^	1.11 ± 0.07 ^b^	0.45 ± 0.02 ^c^	0.109 ± 0.03 ^ab^	9.43 ± 0.21 ^a^
Gel-ga-CNC1	209.14 ± 0.042 ^a^	1.06 ± 0.09 ^c^	0.55 ± 0.05 ^b^	0.116 ± 0.07 ^a^	5.63 ± 0.08 ^b^
Gel-ga-CNC2	237.28 ± 0.015 ^b^	1.05 ± 0.08 ^c^	0.60 ± 0.05 ^a^	0.121 ± 0.08 ^c^	4.00 ± 0.03 ^c^
Gel-ca-CNC1	196.00 ± 0.085 ^b^	1.20 ± 0.03 ^a^	0.44 ± 0.02 ^c^	0.119 ± 0.04 ^a^	8.86 ± 0.39 ^a^
Gel-ca-CNC2	259.143 ± 0.062 ^b^	0.87 ± 0.02 ^d^	0.43 ± 0.03 ^c^	0.117 ± 0.03 ^ab^	6.57 ± 1.34 ^b^

Distinct superscript letters denote statistically significant differences (*p* < 0.05) between the formulations.

**Table 3:** Mean values and associated standard deviations for the mechanical properties of the investigated formulations: Elastic Modulus (EM), Tensile Strength (TS), and Elongation at Break (E).

Formulations	EM (MPa)	TS (MPa)	E (%)
Gel-ga	350 ± 5 ^de^	22.2 ± 0.8 ^e^	93 ± 2 ^ab^
Gel-ca	340 ± 5 ^e^	20.5 ± 0.5 ^f^	98 ± 3 ^a^
Gel-ga-CNC1	366 ± 2 ^c^	28.3 ± 1.0 ^c^	86 ± 2 ^bc^
Gel-ga-CNC2	420 ± 3 ^a^	42.2 ± 2.1 ^a^	83± 2 ^c^
Gel-ca-CNC1	355 ± 3 ^d^	25.0 ± 0.7 ^d^	89 ± 3 ^b^
Gel-ca-CNC2	405 ± 3 ^b^	35.5 ± 0.6 ^b^	85 ± 2 ^bc^

Distinct superscript letters denote statistically significant differences (*p* < 0.05) between the formulations.

**Table 4:** Mean values and associated standard deviations for the melting temperature (Tm) and melting enthalpy (ΔHm) of the studied hydrogels.

Formulations	Tm (°C)	△H_m_ (J/g)
Gel-ga	152.9 ± 0.1 ^e^	271.9 ± 0.5 ^c^
Gel-ca	154.05 ± 0.6 ^d^	304.8 ± 0.5 ^a^
Gel-ga-CNC1	149.7 ± 0.5 ^f^	235.85 ± 0.6 ^e^
Gel-ga-CNC2	169.5 ± 0.5 ^c^	238.1 ± 0.3 ^d^
Gel-ca-CNC1	171.83 ± 0.2 ^b^	212.65 ± 0.5 ^f^
Gel-ca-CNC2	174.6 ± 0.5 ^a^	277.85 ± 0.3 ^b^

Distinct superscript letters denote statistically significant differences (*p* < 0.05) between the formulations.

**Table 5:** Mass fractions of the formulations of the studied hydrogel films.

Formulations	Gelatine	Glycerol	Glutaraldehyde	Citric Acid	CNC
Gel-ga	0.868	0.130	0.025	0.000	0.00
Gel-ca	0.834	0.125	0.00	0.042	0.00
Gel-ga-CNC1	0.849	0.127	0.025	0.000	0.021
Gel-ga-CNC2	0.832	0.124	0.025	0.000	0.041
Gel-ca-CNC1	0.816	0.122	0.00	0.041	0.021
Gel-ca-CNC2	0.801	0.120	0.00	0.040	0.040

## Data Availability

The data presented in this study are available on request from the corresponding author.
